# Trends in Socioeconomic Inequalities in Full Vaccination Coverage among Vietnamese Children Aged 12–23 Months, 2000–2014: Evidence for Mitigating Disparities in Vaccination

**DOI:** 10.3390/vaccines7040188

**Published:** 2019-11-18

**Authors:** Hoang-Long Vo, Le-Thai-Bao Huynh, Hao Nguyen Si Anh, Dang-An Do, Thi-Ngoc-Ha Doan, Thi-Huyen-Trang Nguyen, Huy Nguyen Van

**Affiliations:** 1 Institute for Preventive Medicine and Public Health, Hanoi Medical University, Hanoi 100000, Vietnam; anhhao5896@gmail.com (H.N.S.A.); doanthingocha.hmu@gmail.com (T.-N.-H.D.); 2 Faculty of Medicine, Duy Tan University, Danang 550000, Vietnam; 3 Department of International Cooperation, Ministry of Health, Hanoi 100000, Vietnam; dda.icd@gmail.com; 4 Department of Public Health, Thang Long University, Hanoi 100000, Vietnam; trangnth@thanglong.edu.vn; 5 Graduate School of Public Health, St. Luke’s International University, Tokyo 104-0044, Japan; 6 Department of Quantitative Health Sciences, University of Massachusetts Medical School, Worcester, MA 01655, USA

**Keywords:** full vaccination coverage, socioeconomic inequalities, children aged 12–23 months, Vietnam

## Abstract

There has been no report on the situation of socioeconomic inequalities in the full vaccination coverage among Vietnamese children. This study aims to assess the trends and changes in the socioeconomic inequalities in the full vaccination coverage among Vietnamese children aged 12–23 months from 2000 to 2014. Data were drawn from Multiple Indicator Cluster Surveys (2000, 2006, 2011, and 2014). Concentration index (CCI) and concentration curve (CC) were applied to quantify the degree of the socioeconomic inequalities in full immunization coverage. The prevalence of children fully receiving recommended vaccines was significantly improved during 2000–2014, yet, was still not being covered. The total CCI of full vaccination coverage gradually decreased from 2000 to 2014 (CCI: from 0.241 to 0.009). The CC increasingly became close to the equality line through the survey period, indicating the increasingly narrow gap in child full immunization amongst the poor and the rich. Vietnam witnessed a sharp decrease in socioeconomic inequality in the full vaccination coverage for over a decade. The next policies towards children from vulnerable populations (ethnic minority groups, living in rural areas, and having a mother with low education) belonging to lower socioeconomic groups may mitigate socioeconomic inequalities in full vaccination coverage.

## 1. Introduction

Illness, disability, and death resulting from vaccine-preventable diseases can be prevented by immunization, which is known as putting a substance into the body that makes it produce antibodies [1,2]. The immunization played an important role in achieving one of the aims of the United Nations Millennium Development Goal (MDG) 4, which was to reduce the mortality rate of under-5-year-old children by two-thirds in the 1990–2015 period. The success of the routine immunization program is estimated by vaccination coverage among children aged 12 to 23 months [3]. Global vaccination coverage, which has a proportion of 85% as following the World Health Organization (WHO)’ report, has not changed significantly over the past few years [1]. These estimates suggested that the number of mortalities could be minimized if the potential of existing vaccines was maximized [4].

In Vietnam, the expanded program on immunization (EPI), initiated to be implemented against six target diseases in this country since 1981, was widely expanded at the provincial level nationwide by 1985. After one year, in 1986, the Vietnam EPI was considered one of the six priority national health programs [5,6]. With the great efforts of the EPI, Vietnam had achieved excellent success and had maintained existing achievements in the immunization coverage. In fact, the national vaccination coverage rates in some Southeast Asian countries had decreased as well as significantly fluctuated, in part due to a reduction in funding [7].

After 25 to 30 years of Doi Moi^*^ 1986 (Doi Moi (English: Renovation) is the name given to the economic reforms initiated in Vietnam in 1986 with the goal of creating a “socialist-oriented market economy”. The term ‘Doi Moi’ itself is a general term with wide use in the Vietnamese language), Vietnam achieved great progress for its 26 million children in a remarkably short time [8]. In an effort to achieve Millennium Development Goals (MDGs) to reduce child mortality, Vietnam reduced the under-five mortality rate from 58 per 1000 live births to 24 per 1000 between 1990 and 2009 [9]. Despite this fact, since 2005, the under-five mortality rate in Vietnam has been higher than the average figure for countries in the East Asian and Pacific region [8,10]. Currently, a large number of Vietnamese children still die before they reach their first birthday. Therefore, it is actually important to understand the general trend of the full immunization coverage, individual trends of the recommended vaccines over the years, and issues surrounding vaccination. Little is known about the situation of socioeconomic inequalities in the full vaccination coverage among children in developing countries, especially in Vietnam, a country that remains popular with infectious diseases. Therefore, the aim of this study is to assess the trends and changes in the socioeconomic inequalities in the full vaccination coverage among Vietnamese children aged 12–23 months from 2000 to 2014 based on the most recently available data of the United Nations Children’s Fund (UNICEF).

## 2. Methods 

### 2.1. Data Source

The rounds of the Multiple Indicator Cluster Survey (MICS) were developed and supported by UNICEF to help countries to monitor their children’s and women’s health indicators. The MICS in Vietnam were coordinated by the General Statistics Office (GSO) of Vietnam with support from UNICEF and the United Nations Population Fund (UNFPA). We used raw data from the 2000, 2006, 2011, and 2014 rounds of MICS [11,12,13,14]. In addition to a household questionnaire, questionnaires were administered in each household for women aged 15–49 and children under age five. We extracted data for children aged 12–23 months from the dataset of these four rounds of the survey. In the raw data, the total number of children under five years old was 3346 in MICS 2014, 3678 in MICS 2011, 2680 in MICS 2006, and 3104 in MICS 2000. Of these, we have kept children aged 12–23 months with no missing values recorded. The numbers of children aged 12–23 months in this study were 545 in 2000, 554 in 2006, 760 in 2011, and 785 in 2014 [11,12,13,14].

### 2.2. Variables

Information on the vaccination was collected either from vaccination cards or by asking mothers. All mothers or caretakers were asked to provide vaccination cards. If the vaccination card for a child was available, interviewers copied vaccination information from the cards onto the Vietnam MICS questionnaire. If no vaccination card was available for a child, the interviewer proceeded to ask the mother to recall whether or not the child had received each of the vaccinations and how many doses were received.

#### 2.2.1. Main Outcome Variables

Estimates for the full immunization coverage from four rounds of MICS in Vietnam were based on children aged 12–23 months. The main study outcome variable (or dependent variable) was whether or not 12–23-month-old children fully received all the mentioned vaccinations before a child’s first birthday, which were recommended as the vaccination schedule of the Vietnam EPI in years, 2000, 2006, 2011, and 2014. All vaccinations needed to be received during the first year of life. Definitions of four dependent variables were as follows:

1. *The full vaccination coverage from Viet Nam MICS 2014* was defined that children fully received a BCG vaccination, three doses of DPT vaccination, three doses of Polio vaccination, three doses of Hepatitis B (HepB) vaccination, and three doses of Haemophilus influenzae type b (Hib) vaccination.
2. *The full vaccination coverage from Viet Nam MICS 2011* was defined that children fully received a BCG vaccination, three doses of DPT vaccination, three doses of Polio vaccination, a first dose of measles vaccination, and three doses of HepB vaccination.
3. *The full vaccination coverage from Viet Nam MICS 2006* was defined that children fully received a BCG vaccination, three doses of DPT vaccination, three doses of Polio vaccination, a first dose of measles vaccination, and three doses of HepB vaccination.
4. *The full vaccination coverage from Viet Nam MICS 2000* was defined that children fully received a BCG vaccination, three doses of DPT vaccination, three doses of Polio vaccination, and a first dose of measles vaccination.

#### 2.2.2. Independent Variables

Independent variables included sex (male/female), area (urban/rural), mother’s education (primary or less/lower secondary/upper secondary and tertiary), ethnicity of household head (Kinh/ethnic minority), and wealth index quintiles as detailed below.

### 2.3. Assessment of Socioeconomic Status

The socioeconomic status was estimated by the value of the wealth asset index. The wealth asset index was estimated by using a principal component analysis (PCA). Key variables used in the PCA model were household goods, dwelling characteristics, and the water and sanitation situation along with other household characteristics. The details of the method used for estimating the wealth asset index are described elsewhere [11,12,13,14]. A wealth score was estimated and assigned to each household in the total sample size. The households were ranked by the wealth score and grouped into quintiles ranging from the poorest to the richest.

### 2.4. Measurement of Socioeconomic Inequality

#### 2.4.1. Concentration Index

We calculated the ‘concentration index’ (CCI) to measure the degree of socioeconomic inequality in the full immunization coverage among children aged 12–23 months [15,16]. O’Donnell et al. described the formula for CCI [15] as follows:

(1) $$C=\frac{2}{µ}cov\left(h,r\right).$$

Here, *µ* is the overall percentage of 12–23-month-old children who received full vaccination coverage (before their first birthday), while h represents the values for the full vaccination of each observation, and r indicates the rank of the household socioeconomic status. The CCI of the full vaccination coverage could range between −1 and +1. The CCI takes the value of 0 if the distribution of the full vaccination coverage is completely equal between the children of wealthier and poorer families. If it is negative, it indicates that the concentration of fully receiving vaccinations among children aged 12–23-months before their first birthday is higher among those women in poor families, and if it is positive, it indicates that the concentration of fully receiving vaccination among 12–23-month-old children is higher among those children in wealthier families [15]. Household socioeconomic status was also used in the continuous form to increase the precision of estimation.

#### 2.4.2. Concentration Curve

We also applied the technique of concentration curve (CC) to estimate the degree of the socioeconomic inequalities [15,16]. The CC plots the cumulative percentage of the full vaccination coverage among children aged 12–23 months (on y-axis) against the cumulative percentage of wealth index ranked from the poorest to the richest (on x-axis). The diagonal line from the origin that reflects perfect equality is a linear line at 45° (line of equality). The farther the CC lies above the line of equality, the higher the concentration of the full vaccination coverage among 12–23-month-old children that belong to the poor households. If the CC lies below the 45° line, the full vaccination coverage concentrates among the rich. The further away the CC is from the 45° line, the greater the degree of the socioeconomic inequalities in the full vaccination coverage.

### 2.5. Statistical Analysis

Descriptive analysis was used for estimating the percentage of 12–23-month-old children vaccinated by specific vaccines according to selected characteristics for every round of the MICS survey. We also used descriptive analysis for the figures of the full immunization coverage in each of the MICS rounds. The socioeconomic inequalities in the full vaccination coverage among children aged 12–23 months were measured using the CCI and the CC. We used the Coindex package for the Stata^®^ software to estimate the CCI and to draw the charts. All statistical analyses in this study were conducted by Stata^®^ 15 (StataCorp LLC, USA), using the weighting of variables for women in the dataset. We set the level of statistical significance at 0.05.

### 2.6. Research Ethics

The MICS dataset was available to access after requesting to use it. All users of the data are encouraged to share the research findings. The raw data of four rounds of MICS were obtained with the approval to use the data for this study. The MICS dataset is described in detail on UNICEF’s website (http://mics.unicef.org/). All study participants in the MICS provided informed consent. Individual identifiable information had been removed before the UNICEF made the dataset available to users.

## 3. Results

### 3.1. The Proportion of the Full Vaccination Coverage by Selected Characteristics

As shown in Table 1, the full immunization coverage was higher among Kinh children (53.0%) than minority children (32.5%). These figures for male and female were similar, at 48.5% and 50.8% respectively. Women living in urban areas (58.5%) were more likely to have fully received vaccination than those in rural areas (46.3%). The higher the educational attainment of the mother, the more children aged 12–23 months that received full vaccination. By household’s wealth status from the 1st quintile and 5th quintile, the percentage of children receiving full vaccination before their first birthday was the lowest among the poorest households (33.4%); these figures ranged from 49.3% to 57.9% among richer households.

### 3.2. Trends and Changes in the Vaccination Coverage

**Figure 1:**
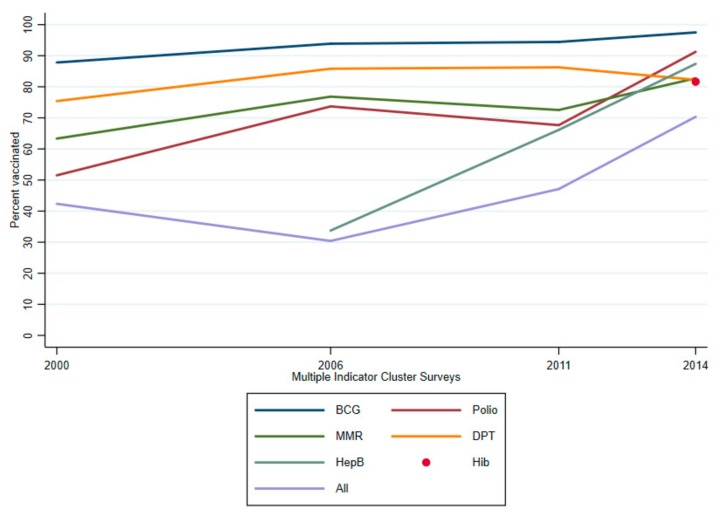
Trends in the coverage of the specific vaccines as well as the full immunization coverage among children aged 12–23 months, 2000–2014.

As shown in Figure 1, there were increasing trends in the percentage of coverage of the specific vaccines as well as the full immunization coverage among children aged 12–23 months. The proportion of the 12–23-month-old children who were fully vaccinated fluctuated between 30.4% and 49.1% in the period 2000 to 2011 before reaching a peak of 70.1% in 2014. In 2000, the proportion of 12–23-month-old children receiving the BCG vaccine was the highest (87.8%), while the figures ranked second for MMR (75.4%) and third for DPT (63.3%). In this same period of 2000, the figure for Polio was the lowest (51.5%). A similar rank for the above vaccines was also observed in the 2006 survey; particularly, the lowest vaccinated proportion was for HepB in the round of 2006 after having just put it on the EPI. This rank remained unchanged in the round of 2011 before the figure for the Polio vaccine dramatically rose and ranked second (91.1%). During the remainder of the period (2011 and 2014), in general, there were significant improvements in the proportion of children who got specific immunizations. It was noticeable for the HepB immunization coverage, which remarkably increased to 87.9% in 2014. The observation for the Hib immunization coverage in children was 81.3% in the round of 2014 after the Hib vaccine was introduced in the EPI in mid-2010.

### 3.3. Trends and Changes in Socioeconomic Inequalities in the Full Vaccination Coverage

**Figure 2:**
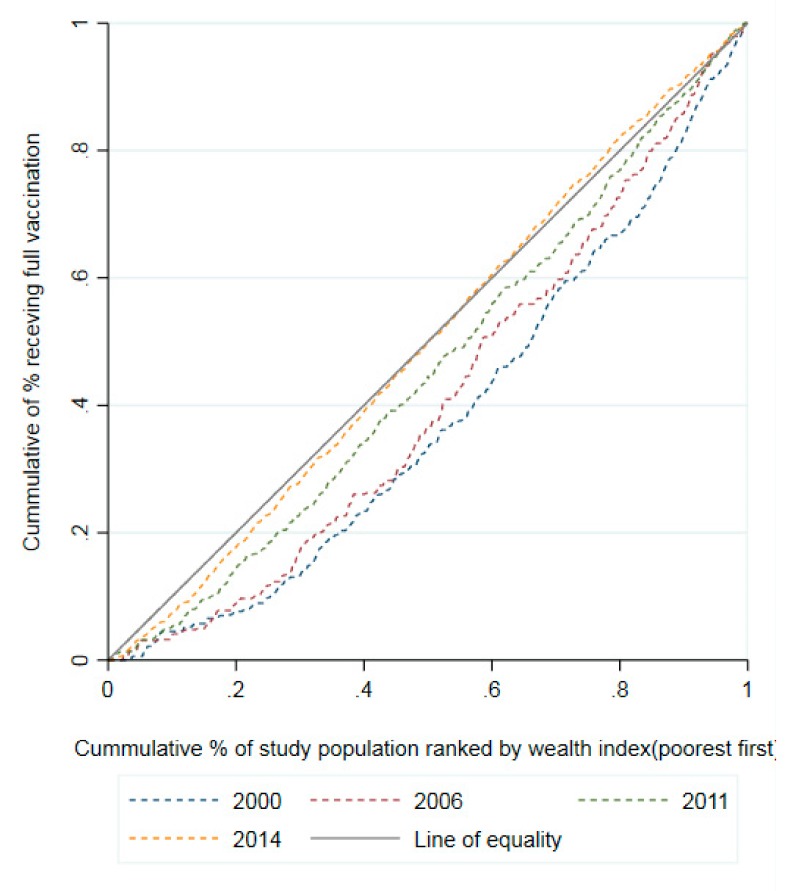
Concentration curves of the full immunization coverage among children aged 12–23 months by wealth index in Vietnam, 2000–2014.

Figure 2 illustrates the CCs of both four years surveyed in full vaccination coverage among 12–23-month-old children. In general, over the years, the CC increasingly became close to the line of equality, which reported that inequality in full vaccination in children was increasingly narrow. However, most CCs were below the inequality line, indicating that the full vaccination coverage among 12–23-month-old children was still concentrated more among the rich. As shown in Table 2, overall, it was clear that the total CCIs of fully receiving vaccination gradually decreased from 2000 to 2014 (CCI: from 0.241 to 0.009), which was statistically significant, except for the 2014 round. By gender and ethnicity, most CCIs of fully receiving vaccination were statistically significantly positive through all four rounds. In particular, the inequalities of receiving full vaccination amongst the poor and the rich were significantly evident in children residing in rural areas and having a mother with primary or less education in all four rounds. Most of the CCIs of full vaccination by selected characteristics had significant reductions between 2000 and 2014, except for children from an ethnic minority group and children having a mother with primary or less education. 

### 3.4. Socioeconomic Factors Associated with the Full Vaccination Coverage

The odds of fully receiving vaccinations among children having a mother with upper secondary and tertiary education was 1.89 (95%CI: 1.46–2.44) times higher than those having women with primary or less education. The odds of fully receiving vaccinations among children belonging to Kinh ethnicity was significantly higher than the odds for those belonging to a minority ethnicity (OR 1.46; 95%CI: 1.02–2.10). Children with better socioeconomic status including poorer, middle, richer, and richest had higher odds ratios of full vaccination coverage than children in the poorest group, at OR 1.52 (95%CI: 1.09–2.14), OR 1.73 (95%CI: 1.21–2.45), OR 1.62 (95%CI: 1.12–2.34), and OR 1.55 (95%CI: 1.06–2.29), respectively (Table 3).

## 4. Discussion

This study was conducted to assess the trends and changes in the socioeconomic inequalities in the coverage of the specific vaccines as well as the full immunization coverage among Vietnamese children aged 12–23 months between 2000 and 2014. We found that, although the percentage of the full immunization coverage significantly fluctuated between 30.4% and 49.1% (from 2000 to 2006), this figure for the round 2014 had a dramatic growth to 70.1%. This increasing achievement may be mainly explained by the efforts of the Vietnam EPI, especially with support from the Global Alliance for Vaccines and Immunization (GAVI) since 2003 [6,11,12,14]. Furthermore, in the period from 2008 to 2016, the investment from the state budget for the health sector always reached 7–8% of the GDP, and the increased rate of budget support every year was higher than those of other sectors. It was not enough evidence for us to fundamentally discuss vaccination trends compared to spending on publicity for a vaccination program. Nevertheless, based on the overall picture of Vietnam’s growing socioeconomic context in recent years, changes in public spending for vaccination programs may have a significant impact on child immunization trend in Vietnam (because it is crucial to understand that the recommended vaccines for children in the Vietnam EPI schedule be free). As was shown in the present results, the socioeconomic inequalities in the full immunization coverage sharply decreased as demonstrated by decreased concentration indices from 0.24 in 2000 to 0.01 in 2014. However, the inequalities between the poor and the rich still emergently remained in the full immunization coverage as measured by gender, region, women’s wealth, living area, and ethnicity. Interestingly, the study’s results highlight the substantial increase in the coverage of full immunization among 12–23-month-old children in this country. The present study not only sheds light on the decreasing trend in socioeconomic inequality in all surveys but also provided important insights for the health system regarding the need for specific actions aimed at addressing the remaining socioeconomic inequalities in the full immunization coverage as the uptake of the immunization services for all children in Vietnam.

In this study, we found that the lowest vaccinated proportion among children was recorded for HepB in the round of 2006. In Vietnam, the HepB vaccine was initiated to be introduced in EPI in 1997. Due to the limited production capacity of the local vaccine manufacturer, the vaccine was only used in a limited number of districts in Hanoi Capital and Ho Chi Minh City [17]. Until 2000, the number of provinces implementing the HepB immunization for children under five years old was 42 out of 60 provinces nationwide [17]. The low coverage of the HepB vaccination from the data of MICS in our study might be due to the fact that children vaccinated against HepB at birth officially started in the EPI in the whole of Vietnam in 2005 [18], hence, it was difficult to gain a full coverage throughout the country after only one year. According to Vietnam EPI report, the vaccination rate for HepB vaccine within 24 h after birth remained very low in three years after (2007, 2008, and 2009), which might be partly due to either the difficult uptake of HepB vaccination in the birth facilities located in remote rural areas or the locals’ acceptance from different ethnic groups. Noticeably, we recorded a positive figure for HepB immunization coverage, which remarkably increased to 87.9% in 2014. This result was considered as an initial success of the Vietnam EPI in a short time, suggesting that the management and administration of the vaccination were step-by-step improved together with effective coordination and use of domestic resources and foreign assistance.

Our finding indicates that a similar rank between all vaccines remained basically unchanged for five years (2006 to 2011), before the figure for the Polio vaccine immunization, which displays an upward trend, and then ranked second in 2014 (91.1%). Our finding was completely consistent when the available information reported that Vietnam initiated the implementation of the National Plan for Poliomyelitis Eradication in 1991 and succeeded in eliminating Polio in 2000 [17,19]. In addition, during the remainder of the period surveyed, overall, there were significant improvements in the proportion of children who got specific immunizations before a child’s first birthday. In particular, the high figure for Hib immunization coverage in children (81.3%) in the round of 2014 was observed after the Hib vaccine was introduced in the EPI in mid-2010. In the Western Pacific region, the WHO regional office has set immunization milestones for maintaining Polio eradication, maternal and neonatal tetanus elimination, measles elimination, and for further reducing HBsAg prevalence among five-year-old children to less than 1% by 2017. We found that more and more increasing prevalence of full immunization coverage in children before their first birthday will certainly contribute to the success in the uptake of adequate vaccination among five-year-old children. Vietnam has been working hard to obtain high coverage rates for most recommended vaccines in the EPI to maintain Polio eradication and neonatal tetanus elimination and to head toward measles elimination and control of HepB [20].

Previously available research evidence indicated an association between the characteristics of the children and their circumstances as factors that deter people from utilizing timely vaccination service [21]. Studies from low income and lower middle-income countries also found that income was considered as an important determinant of the uptake of vaccination services during the first year of life [5,22,23]. In our study, a significant variation between 2000 and 2014 was recorded for the full immunization coverage amongst children belonging to richer households and those from poorer households, suggesting that richer families were more likely to fully vaccinate their children compared to poorer families, which was consistent with previous findings in Bangladesh [24] and Ethiopia [25]. This finding indicates that poverty is a key influencing factor for the use of Vietnam vaccination services during the time surveyed. Our results are in line with other studies in Vietnam and elsewhere, which reported that the inequality existed amongst children from poor households and others from the rich households [23,26,27]. Lower full immunization coverage in poorer households might be explained because of (1) a negative attitude toward the acceptability of vaccination for a child, (2) remote living areas where there is difficulty in making free immunization available, and (3) limited freedom of decision making. Interestingly, overall, we did not find the existent inequality in full immunization coverage amongst the poor and the rich in the 2014 round. This successful result is considered as a persistent effort of Vietnam EPI in maintaining and perfecting the network of specialized staff to EPI from central levels to grassroots levels; thereby, ensuring all recommended free vaccines for both rich and poor children are met.

Vietnam is known as a multi-ethnic nation (more than 53 ethnic groups), and each ethnic group has symbolic cultural characteristics [28]. In the current study, ethnicity also emerged as a key factor existing as the inequality amongst the poor and the rich in the completion of the full immunization before a child’s first birthday. Our finding was consistent with the previous reports, indicating that the gap of the inequality amongst the poor and the rich in the completion of full vaccination was higher among ethnic minority groups than among the Kinh group [21,29]. In addition, we also found that the inequality in the full immunization coverage amongst the poor and the rich was concentrated among children having mothers with primary or less education over the years. It is also noteworthy that during the period of more than 10 years, there were no significant changes in full immunization coverage between the rich and the poor among ethnic minority groups, and among the group of children having mothers with primary or less education. In fact, ethnic minority groups in Vietnam reside mostly in disadvantaged locations with a backward economy, so there are barriers to going to school, and few people can complete 12/12-year educational level according to Vietnamese education regulation [30]. The differences in the uptake of full vaccination between the poor and the rich among Vietnamese children from various ethnic minority groups and having mothers with low education might be because of the differential levels of availability as well as access to full immunization coverage in their residing places [31]. Another explanation could be cultural beliefs behind the acceptance of vaccination services among the child’s mother and their family [32].

Previous evidence in Vietnam studies and elsewhere suggested the residence area as a factor associating with higher coverage of full immunization [21,29]. We found that the inequality still existed between the children of wealthier and poorer families among rural areas for all the time surveyed, 2000–2014, whereas no significant gap between poor families and rich families was observed in children residing in urban areas. One possible explanation for this finding might lie in the increased availability and quality of vaccination services in the country’s urban areas through the concentrated efforts by the government and private organizations.

Our study was based on the MICS dataset, having a nationally representative nature with multiple objectives as well as large sample sizes from four different rounds throughout the survey periods. These enabled us to assess the trend in socioeconomic inequality in full vaccination coverage over a long time on the basis of this country’s long-term healthcare development strategies. However, several limitations of this study need acknowledging. First, due to the foundation of cross-sectional research design from MICS, causality between full vaccination and selected socioeconomic factors could not be determined. Second, the estimations of full immunization coverage were derived from available information on vaccination cards and reported by mothers if their children got each of the vaccinations and how many doses were received; therefore, there exists the possibility that some children might have been vaccinated, yet did not have immunization cards and were excluded from the study. In addition, mothers may have forgotten to report the vaccinations of their children during their interviews. These would underestimate the full immunization coverage in this study. Third, another limitation of this study was our inability to assess the cultural aspects related to the coverage of full immunization, such as acceptability and attitudes of Vietnamese women having children aged 12–23 months towards the uptake of full immunization services. Cultural beliefs and preferences of women and their families from different backgrounds may affect their vaccination service-seeking behavior and may function as a confounding factor or an interacting variable in the assessment of the association between other independent variables and the coverage of full vaccination. Furthermore, religious beliefs might be one of the factors contributing to poor vaccination trends in certain population groups, particularly for a multi-religious country like Vietnam; however, this perspective was not available in the scope of the MICS data. Fourth, because of the limitations of the MICS cross-sectional study design, the immunization data could not be assessed sequentially by month for the current outcome variables (full vaccination coverage) in the four survey rounds. In addition, the outcome variables were obtained from multiple vaccines recommended within the first year after birth, so the evaluation of data looking at the month was not likely to be performed. Finally, the data on infectious pathogens were not included in MICS; therefore, the effect of the vaccination on the spread of infectious pathogens on different socioeconomic groups was unknown.

Despite these weaknesses, the findings in the current study contain meaningful policy implications that need to be noted. First, this study confirmed that there were some trade-offs between the increase in full immunization coverage and the reduction of socioeconomic inequalities. Second, vulnerable population groups are identified, which provides the policy makers with vital information to reach target populations effectively; thereby, equitably and sustainably tackling the inequalities in the full vaccination. Third, changes in the vaccination service-seeking behavior might be maximized by mass campaign messages based on evidence of the vulnerable populations found in immunization coverage. In addition to this, campaign messages should be tested during such campaign development.

## 5. Conclusions

The current study suggests a sharp reduction in socioeconomic inequalities in the full vaccination coverage among 12–23-month-old Vietnamese children over a decade, from 2000 to 2014. The higher socioeconomic groups tend to achieve EPI-related immunization more actively than their lower socioeconomic counterparts. We highlighted the need for follow-up studies to explore the barriers towards the uptake as well as the use of vaccination services in the Vietnam EPI, such as attitude and acceptability for vaccination in different ethnic groups. We recommend implementing specific priority interventions for children from ethnic minority groups, living in rural areas, and having a mother from low education for better outcomes in full immunization; from that, gradually taking to the equal distribution of child vaccination and beyond, promoting long-lasting herd immunity in Vietnam. The findings of this study once again confirmed the important role of immunization that was given as one of four main strategies in the target for the Millennium Development Goal (MDG) 4 (reduce the mortality rate of children under five years old by two-thirds between 1990 and 2015).

## Figures and Tables

BCG = Bacillus Calmette–Guérin vaccine; DPT = tetanus–diphtheria–pertussis vaccine; MMR = Measles–Mumps–Rubella vaccine; HepB = hepatitis B vaccine; Hib = Haemophilus influenzae type b vaccine.

**Table 1:** Proportion of children aged 12–23 months who were vaccinated by specific vaccines in Vietnam by selected characteristics in the four rounds of Multiple Indicator Cluster Survey (MICS) in Vietnam, 2000–2014.

Characteristics	Percentage of 12–23-Month Children Received						
	BCG	Polio	DPT	MMR	HepB	Hib	Full
	% (SE)	% (SE)	% (SE)	% (SE)	% (SE)	% (SE)	% (SE)
Sex							
Male	93.9 (0.9)	71.7 (1.7)	73.3 (1.6)	82.8 (1.4)	62.9 (2)	80.2 (2.2)	48.5 (1.8)
Female	93.9 (0.9)	74 (1.9)	76.1 (1.5)	82.7 (1.3)	68.6 (1.9)	83.2 (2.2)	50.8 (1.9)
Area							
Urban	98.2 (0.5)	82.4 (1.6)	84.4 (1.7)	84.8 (1.5)	77.6 (2.1)	82.8 (2.5)	58.5 (2.2)
Rural	92.3 (0.9)	69.2 (2)	71 (1.6)	82 (1.3)	60.7 (2.1)	81.1 (2.2)	46.3 (1.8)
Mother’s Education							
Primary or Less	86.8 (1.6)	57.9 (2.8)	62.6 (2.6)	74.8 (2.2)	53.1 (2.9)	75.5 (4.2)	38.5 (2.3)
Lower Secondary	95.2 (0.8)	74.1 (2)	75.6 (1.7)	85.1 (1.3)	62.1 (2.4)	79.4 (3)	48.3 (2.1)
Upper Secondary and Tertiary	98.2 (0.5)	83.6 (1.5)	83.6 (1.5)	86.7 (1.3)	77.2 (1.9)	85.6 (2.2)	60.4 (2)
Ethnicity							
Kinh	96.3 (0.5)	76.7 (1.5)	79.4 (1.2)	85.7 (1)	69.1 (1.7)	84.1 (1.8)	53.0 (1.5)
Minority	81.7 (2.5)	52.7 (3.7)	50.7 (3.5)	68.2 (3.1)	46.6 (4.2)	67.7 (4.8)	32.5 (3.4)
Mother’s Wealth Index							
1st Quintile (Poorest)	83.7 (2.2)	53.6 (3.2)	55.9 (2.9)	68.9 (2.8)	49 (3.6)	72.5 (4.4)	33.4 (2.8)
2nd Quintile	94.4 (1.1)	72.1 (2.4)	72.2 (2.4)	85.3 (1.7)	64.1 (3.3)	77.8 (3.8)	49.3 (2.7)
3rd Quintile	96.8 (1)	74.9 (2.7)	78.1 (2.2)	89.3 (1.5)	67.4 (3)	85 (3.2)	54.1 (2.9)
4th Quintile	97.3 (0.8)	80.5 (2.3)	81.6 (2.2)	86.3 (1.8)	69.9 (3)	85.8 (3.5)	55.2 (2.8)
5th Quintile (Richest)	98.5 (0.6)	84.8 (1.8)	87.5 (1.7)	86 (1.8)	76.3 (2.7)	85.6 (3.2)	57.9 (2.7)

BCG = vaccination to protect against tuberculosis; Polio = vaccination to protect against polio; DPT = vaccination to protect against diphtheria, pertussis, tetanus; MMR = vaccination to protect against measles, mumps, and rubella; HepB = vaccination to protect against hepatitis B; Hib = vaccination to protect against Haemophilus influenzae type b.

**Table 2:** Concentration indices of fully receiving vaccinations among children aged 12–23 months in Vietnam by different social factors, 2000–2014.

Characteristics	Concentration Index of Fully Receiving Vaccinations				Change from 2000 to 2014 (SE)
	2000	2006	2011	2014	
Sex					
Male	0.233 *** (0.045)	0.124 * (0.061)	0.05 (0.038)	0.04 * (0.019)	−0.193 *** (0.049)
Female	0.249 *** (0.037)	0.245 *** (0.052)	0.128 ** (0.039)	−0.026 (0.025)	−0.275 *** (0.045)
Area					
Urban	0.092 (0.047)	−0.057 (0.058)	0.002 (0.035)	−0.023 (0.032)	−0.114 * (0.057)
Rural	0.239 *** (0.048)	0.189 ** (0.064)	0.081 ** (0.042)	0.051 ** (0.019)	−0.188 *** (0.051)
Ethnicity					
Kinh	0.167 *** (0.029)	0.107 * (0.044)	0.06 * (0.029)	−0.024 (0.017)	−0.191 *** (0.033)
Minority	0.275 * (0.123)	0.121 (0.203)	0.233 ** (0.087)	0.136 *** (0.035)	−0.138 (0.128)
Mother’s Education					
Primary or Less	0.213 ** (0.074)	0.285 *** (0.062)	0.272 *** (0.073)	0.147 *** (0.034)	−0.066 (0.081)
Lower Secondary	0.126 ** (0.044)	0.161 * (0.07)	0.058 (0.044)	0.01 (0.026)	−0.116 * (0.051)
Upper Secondary and Tertiary	0.081 (0.054)	0.027 (0.085)	−0.016 (0.038)	−0.045 * (0.023)	−0.126 * (0.059)
Total	0.241 *** (0.031)	0.182 *** (0.044)	0.089 ** (0.031)	0.009 (0.017)	−0.232 *** (0.035)

CCI: concentration index; SE: standard error; *, **, ***: significant at 0.05, 0.01 and 0.001 (paired t-test to compare the concentration index between two rounds).

**Table 3:** Full vaccination coverage among Vietnamese children aged 12–23 months and associated socioeconomic factors between 2000 and 2014: multivariable logistic regression analysis.

Characteristics	12–23-Month-Children Fully Receiving Vaccinations Between 2000 and 2014
	OR (95% CI)
Mother’s Education	
Primary or Less	1
Lower Secondary	1.25 (0.98–1.58)
Upper Secondary and Tertiary	1.89 *** (1.46–2.44)
Ethnicity	
Minority	1
Kinh	1.46 * (1.02–2.10)
Mother’s Wealth Index	
1st Quintile (Poorest)	1
2nd Quintile (Poorer)	1.52 * (1.09–2.14)
3rd Quintile (Middle)	1.73 ** (1.21–2.45)
4th Quintile (Richer)	1.62 * (1.12–2.34)
5th Quintile (Richest)	1.55 * (1.06–2.29)

OR: odds ratio; CI: confidence interval; *, **, ***: significant at 0.05, 0.01, and 0.001
